# Identification of Salt-Stress-Responding Genes by Weighted Gene Correlation Network Analysis and Association Analysis in Wheat Leaves

**DOI:** 10.3390/plants13182642

**Published:** 2024-09-21

**Authors:** Linyi Qiao, Yijuan Li, Liujie Wang, Chunxia Gu, Shiyin Luo, Xin Li, Jinlong Yan, Chengda Lu, Zhijian Chang, Wei Gao, Xiaojun Zhang

**Affiliations:** 1 College of Agronomy, Shanxi Key Laboratory of Crop Genetics and Molecular Improvement, Shanxi Agricultural University, Taiyuan 030031, China; linyi.qiao@sxau.edu.cn (L.Q.); liyijuan111@126.com (Y.L.);; 2 Millet Research Institute, Shanxi Agricultural University, Changzhi 046011, China

**Keywords:** wheat, leaf, salt stress, WGCNA, candidate genes

## Abstract

The leaf is not only the main site of photosynthesis, but also an important organ reflecting plant salt tolerance. Discovery of salt-stress-responding genes in the leaf is of great significance for the molecular improvement of salt tolerance in wheat varieties. In this study, transcriptome sequencing was conducted on the leaves of salt-tolerant wheat germplasm CH7034 seedlings at 0, 1, 6, 24, and 48 h after NaCl treatment. Based on weighted gene correlation network analysis of differentially expressed genes (DEGs) under salt stress, 12 co-expression modules were obtained, of which, 9 modules containing 4029 DEGs were related to the salt stress time-course. These DEGs were submitted to the Wheat Union database, and a total of 904,588 SNPs were retrieved from 114 wheat germplasms, distributed on 21 wheat chromosomes. Using the R language package and GAPIT program, association analysis was performed between 904,588 SNPs and leaf salt injury index of 114 wheat germplasms. The results showed that 30 single nucleotide polymorphisms (SNPs) from 15 DEGs were associated with salt tolerance. Then, nine candidate genes, including four genes (*TaBAM*, *TaPGDH*, *TaGluTR*, and *TaAAP*) encoding enzymes as well as five genes (*TaB12D*, *TaS40*, *TaPPR*, *TaJAZ*, and *TaWRKY*) encoding functional proteins, were identified by converting salt tolerance-related SNPs into Kompetitive Allele-Specifc PCR (KASP) markers for validation. Finally, interaction network prediction was performed on *TaBAM* and *TaAAP*, both belonging to the Turquoise module. Our results will contribute to a further understanding of the salt stress response mechanism in plant leaves and provide candidate genes and molecular markers for improving salt-tolerant wheat varieties.

## 1. Introduction

It is expected that by 2050, 50% of the global arable land will be affected by salinization, posing a severe challenge to agricultural production [1]. Wheat (*Triticum aestivum* L.) is the most widely planted crop in the world. Improving the tolerance of wheat varieties to saline soil and achieving stable and high yields is of great significance for ensuring food security and sustainable agricultural development. Throughout the entire growth cycle of wheat, the seedling stage is the most sensitive to salt [2]. High salt concentrations in soil cause osmotic stress on seedling roots, while Na^+^ enters root cells through non-selective cation channels and is transported to leaves through xylem, thereby affecting photosynthesis and physiological and biochemical reactions [3,4].

Exploring salt tolerance genes from roots and leaves can be used for improving salt tolerance in wheat varieties. For wheat, a heterozygous hexaploid with a large and complex genome [5], weighted gene co-expression network analysis (WGCNA) based on transcriptome sequencing data is a fast and effective method for mining salt-tolerant candidate genes. Due to the fact that roots are the first organ in plants to perceive soil salinity, the transcription of a large number of root genes can be induced by salt stress. Li et al. [6] conducted WGCNA analysis on the root transcriptome data of wheat salt-tolerant line ST9644 under salt stress at 0, 6, and 24 h, obtaining four response modules and identifying nine salt-tolerant candidate genes, of which five candidates encode ATP binding cassette (ABC) transporters. Using a similar method, Chen et al. [7] obtained four response modules from the root transcriptome data of wheat salt-tolerant line CH7034 under salt stress at 0, 1, 6, 24, and 48 h, and identified four salt-tolerant candidate genes encoding glutathione S-transferase (TaGST), Walls Are Thin 1 (WAT1)-related protein (TaWAT), zinc finger protein (TaZFP), and aquaporin (TaAQP), respectively.

Leaves are the main site of photosynthesis and an important organ reflecting salt tolerance of plants. Several genes in wheat leaves, such as U-box E3 ubiquitin ligase gene *TaPUB1* [8], histone acetyltransferase gene *TaHAG1* [9], and abscisic acid-stress-ripening gene *TaASR1-D* [10], have been confirmed to maintain a normal Na^+^ concentration in the cytoplasm, decrease the degradation of chlorophyll in chloroplast, and reduce salt-induced reactive oxygen species (ROS) and oxidative damage to the plasma membrane under stress conditions. However, research on the systematic exploration of salt-tolerant genes from wheat leaves has not yet been reported. Here, we performed RNA-Seq on the leaves of wheat salt-tolerant line CH7034 during the seedling stage under NaCl stress at 0, 1, 6, 24, and 48 h, and used WGCNA to identify salt-stress-responding modules. Then, association analysis was conducted based on leaf salt injury index data from a set of wheat germplasm to confirm salt-tolerance candidate genes.

## 2. Results

### 2.1. Transcriptional Response to Salt Stress in Wheat Leaves

**Figure 1:**
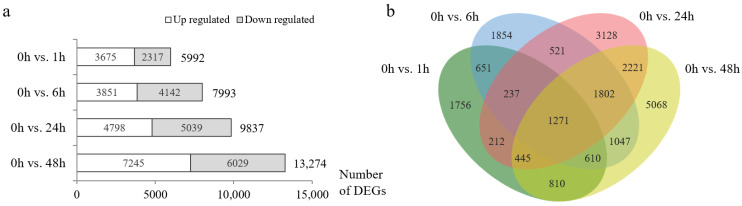
Differentially expressed genes (DEGs) under salt stress in the leaves of wheat strain CH7034. (**a**) The number of DEGs at 4 timepoints after 250 mmol/L NaCl treatment. (**b**) Venn diagram of DEGs.

The transcriptome sequencing of 15 leaf samples of the salt-tolerant line CH7034 resulted in 174.80 Gb clean reads. Each sample obtained clean data above 9.54 Gb with Q30-based percentages greater than 93.23% and GC percentages ranging from 53.39 to 54.79% (Table S1), indicating that the RNA-Seq data were of high quality for further analysis. Subsequently, 584,308,552 clean reads were mapped on the Chinese Spring reference genome RefSeq v1.0, and the uni-transcripts were annotated as known genes. A total of 50,106 genes with an average fragments per kilobase of exon model per million mapped fragments (FPKM) of >1 in at least one treatment (1, 6, 24, and 48 h) were considered expressed genes. Differential expression analysis identified 5992 (3675 upregulated/2317 downregulated), 7993 (3851 upregulated/4142 downregulated), 9837 (4798 upregulated/5039 downregulated), and 13,274 (7245 upregulated/6029 downregulated) differentially expressed genes (DEGs) at 1, 6, 24, and 48 h of NaCl treatment, respectively, compared with the transcriptome data at 0 h (Figure 1a). Overall, 13,435 DEGs were upregulated or downregulated in at least one treatment, and 4017 DEGs appeared at each stress timepoint (Figure 1b).

### 2.2. Co-Expression Modules of Salt-Stress-Responding Genes

**Figure 2:**
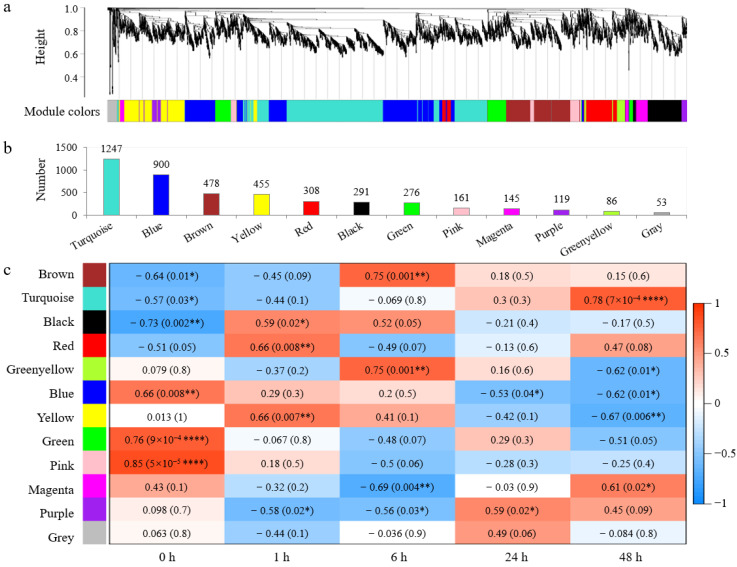
Weighted gene co-expression network analysis (WGCNA) for DEGs responding to salt stress in wheat leaves. (**a**) Cluster dendrogram and module colors of 4519 DEGs. (**b**) The number of DEGs contained in each module. (**c**) Heatmap for the relationships of modules and salt treatment time-courses. Each cell lists the correlation index and *p*-value in parentheses; * indicates *p* < 0.05, ** indicates *p* < 0.01, and **** indicates *p* < 0.0001.

WGCNA was performed on the 13,435 DEGs screened above, and 4519 genes were clustered and divided into 12 modules with different colors based on their expression patterns (Figure 2a, Table S2). Among them, the Turquoise module contains the most DEGs (1247), while the Grey module contains the fewest DEGs (53) (Figure 2b).

Correlation analysis results showed that (1) the expression patterns of four modules were significantly correlated with one timepoint of salt stress treatment: the Black module and Red module were significantly positively correlated with 1 h (*p* < 0.05), the Brown module was extremely significantly positively correlated with 6 h (*p* < 0.01), and the Turquoise module was extremely significantly positively correlated with 48 h (*p* < 0.01); (2) the expression patterns of four modules were significantly correlated with two timepoints of salt stress treatment: the Green-yellow module was highly positively correlated with 6 h (*p* < 0.01) and negatively correlated with 48 h (*p* < 0.05), the Blue module was significantly negatively correlated with 24 h and 48 h (*p* < 0.05), the Yellow module was highly positively correlated with 1 h (*p* < 0.01) and negatively correlated with 48 h (*p* < 0.01), and the Magenta module was highly negatively correlated with 6 h (*p* < 0.01) and 48 h (*p* < 0.05); (3) the expression pattern of the Purple module was significantly correlated with three timepoints of salt stress treatment: significantly negatively correlated with 1 h and 6 h (*p* < 0.05), and significantly positively correlated with 24 h (*p* < 0.05); (4) in addition, the Green module and Pink module were not correlated with the timepoints after salt treatment (1, 6, 24, and 48 h), while the Gray module was not correlated with all timepoints (Figure 2c).

### 2.3. Association Analysis between DEGs and Leaf Salt Injury Index

**Figure 3:**
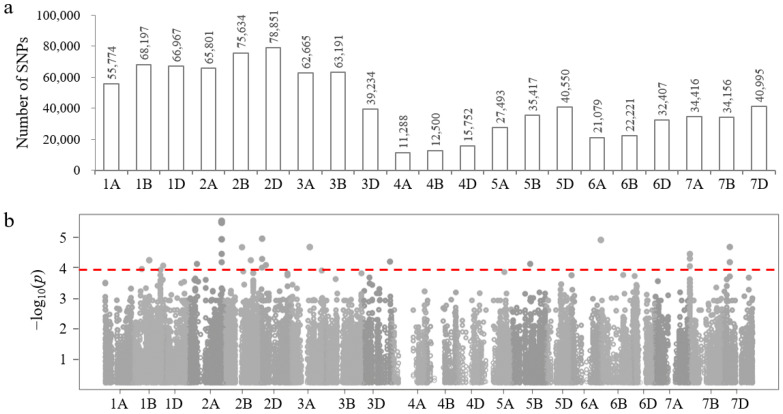
Association analysis between salt-stress-response-module genes and salt-tolerance phenotype of wheat leaves. (**a**) Chromosome distribution of SNPs derived from target module genes. (**b**) Manhattan plot of association analysis between SNPs and leaf salt injury index of 114 wheat germplasms. The threshold is set to −log_10_ (*p*) > 4.

Except for Gray, Green, and Pink modules, the expression patterns of the other nine modules are correlated with the time-course of salt stress. The 4029 DEGs contained in these nine modules were submitted to the Wheat Union database, and then a total of 904,588 SNPs distributed on 21 wheat chromosomes were obtained from 114 wheat germplasms. Among them, chromosome 2D had the most, 78,851 SNPs, while chromosome 4A had the least, 11,288 SNPs (Figure 3a).

Association analysis was conducted between these 904,588 SNPs and the leaf salt injury index (LSI) of 114 wheat germplasms, and the results showed that 30 SNPs from chromosomes 1B, 2A, 2B, 2D, 3A, 3D, 5B, 6B, 7B, and 7D were correlated with the salt-tolerance phenotype (−log_10_ *p* > 4) (Figure 3b, Figure S1).

### 2.4. DEGs Responding to Salt Stress in Leaves

The 30 SNPs selected through association analysis come from 15 genes belonged to eight modules: the four genes from the Yellow module, three genes from the Turquoise module, two genes from the Brown module, two genes from the Red module, and each has one gene from the Purple, Blue, Black, as well as Magenta modules (Table 1). Among them, *TraesCS2A02G508900* has five SNPs related to salt tolerance, *TraesCS3A02G223700* and *TraesCS7B02G024500* each have four salt-tolerance-related SNPs, *TraesCS7D02G189000* has three salt-tolerance-related SNPs, *TraesCS1B02G199700*, *TraesCS2D02G073700*, and *TraesCS6B02G031700* each have two salt-tolerance-related SNPs, and the remaining eight genes each have one salt-tolerance-related SNP.

### 2.5. KASP Marker Validation of Candidate Genes

**Figure 4:**
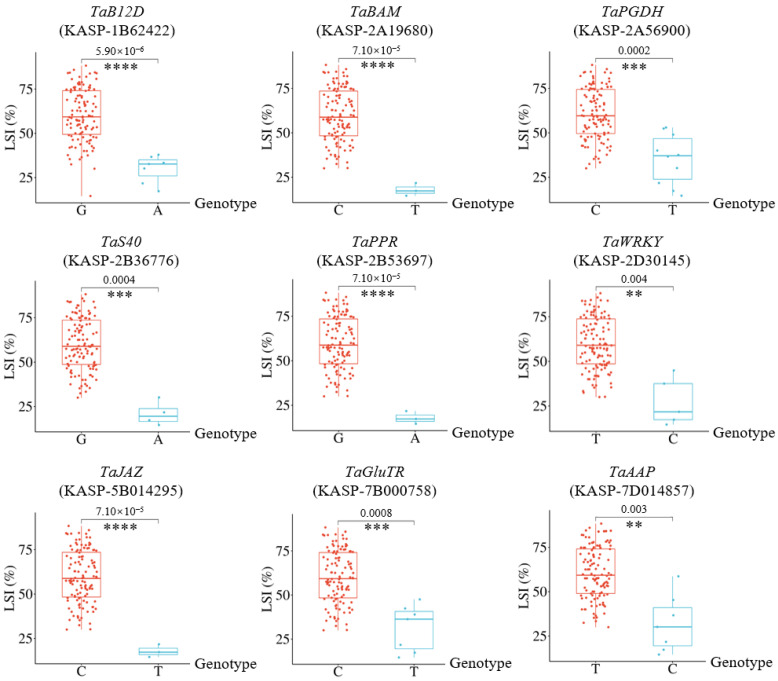
KASP validation of candidate genes. LSI: leaf salt injury index; the bases listed on horizontal axis represent the binary genotypes of each KASP marker. ** indicates *p* < 0.01, *** indicates *p* < 0.001, and **** indicates *p* < 0.0001.

To validate the results of association analysis and provide molecular markers for breeding, the SNPs of 15 salt-tolerant candidate genes mentioned above were transformed into Kompetitive Allele-Specific PCR (KASP) markers for testing in 114 wheat germplasms, and the expected genotyping results were obtained for nine genes (*p* < 0.01) (Figure 4). Among them, four genes encode enzymes, including *TaBAM* (*TraesCS2A02G215100*) encoding β-amylase, *TaPGDH* (*TraesCS2A02G508900*) encoding 3-phosphoglycerate dehydrogenase, *TaGluTR* (*TraesCS7B02G024500*) encoding glutamyl-tRNA reductase, and *TaAAP* (*TraesCS7D02G18900*) encoding amino acid permease; the remaining five genes encode different types of proteins, including *TaB12D* (*TraesCS1B02G433700*) encoding B12D protein, *TaS40* (*TraesCS2B02G286300*) encoding senescence regulator S40, *TaPPR* (*TraesCS2B02G410100*) encoding pentatricopeptide repeat-containing protein, *TaJAZ* (*TraesCS5B02G211000*) encoding jasmonate zim-domain protein, and *TaWRKY* (*TraesCS2D02G168600*) encoding WRKY transcription factor.

### 2.6. Prediction of the Interaction Network between TaBAM and TaAAP

**Figure 5:**
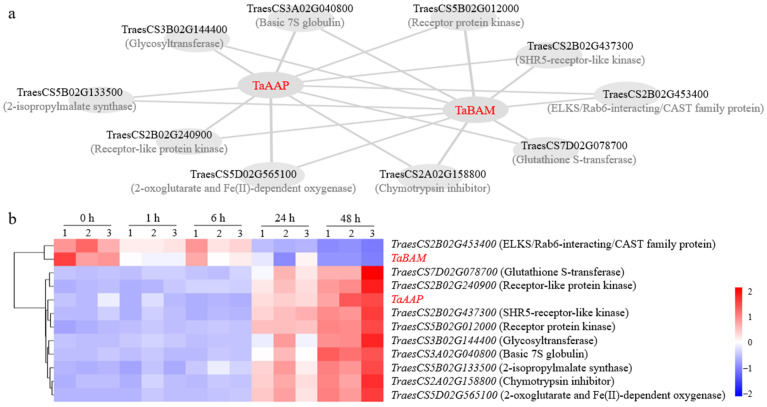
Predictive interaction network for *TaBAM* and *TaAAP* (**a**) and their transcriptional response to salt stress (**b**).

*TaBAM* and *TaAAP* belong to the Turquoise module with the most DEGs. We selected DEGs with edge weights >0.4 from this module to construct an interaction network for *TaBAM* and *TaAAP*. The results showed that 10 DEGs, including seven genes encoding biochemical reaction enzymes and three genes encoding functional proteins, were predicted as the interacting genes of *TaBAM* and *TaAAP* (Figure 5a). The transcriptome results showed that *TaBAM* and *TraesCS2B02G453400* encoding ERC protein had similar expression patterns and were significantly downregulated at 48 h after salt stress, while *TaAAP* was grouped with the other nine genes and significantly upregulated 48 h after salt stress (Figure 5b).

## 3. Discussion

### 3.1. Modules Responding Salt Stress in Wheat Leaves

The response of plant cells to salt stress can be roughly divided into four phases: early signal transduction phase (with 5 min), stop phase (5 min~5 h), quiescent phase (5~9 h), and growth recovery phase (9 h~) [4]. In this study, we set four salt stress timepoints: 1 h for stop phase, 6 h for quiescent phase, 24 and 48 h for growth recovery phase. The RNA-Seq results showed that the number of DEGs in the leaves increased with prolonged treatment time compared to control (0 h). Using WGCNA analysis, nine co-expression modules related to the time-course of salt stress were identified. Among them, four modules including Black, Red, Yellow, and Purple responded significantly during the stop phase (1 h), four modules including Brown, Green-yellow, Magenta, and Purple responded significantly during the quiescent phase (6 h), and five modules including Turquoise, Blue, Green-yellow, Yellow, and Magenta responded significantly during the growth recovery phase (24 and 48 h). Similar to the RNA-Seq results, the number of responded modules increased in the later phase of salt treatment.

### 3.2. Salt-Tolerant Candidate Genes

Through WGCNA, association analysis, and KASP validation, nine salt-tolerant candidate genes were identified from responded modules. Among them, four genes including *TaPGDH*, *TaGluTR*, *TaBAM*, and *TaAAP* encoding enzymes. Previous studies have shown that PGDH [11], GluTR [12], BAM [13], and AAP [14] involved salt tolerance in plants.

Moreover, the remaining five genes encode functional proteins or transcription factors. *TaJAZ* encodes the wheat JAZ family member JAZ9-B [15]. It was reported that the expression level of *JAZ9-B* in the seedling leaves of wheat breeding line BS366 continued to decrease after 4 h of NaCl treatment [15], which is consistent with the change trend of the Yellow module containing *TaJAZ* in this study. *TaWRKY* encodes the wheat transcription factor WRKY34-D [16], and its family member WRKY75-A has been confirmed to enhance salt tolerance of overexpressing plants [16]. *TaB12D* encodes B12D protein. In rice seedling, *OsB12D1* is induced by salt stress [17], while in wheat seedling roots, a *B12D* gene is significantly induced in response to water deficiency stress [18], indicating that B12D protein is involved in abiotic stress responses. In addition, S40 protein [19] and PPR protein [20], similar products encoded by *TaS40* and *TaPPR*, have also been reported associated with salt tolerance in wheat. Next, we will further investigate the functional mechanisms of these nine candidate genes in the salt stress response, and their related KASP markers can be used for germplasm evaluation or molecular assisted breeding.

### 3.3. Predicted Interaction Network in Turquoise Module

**Figure 6:**
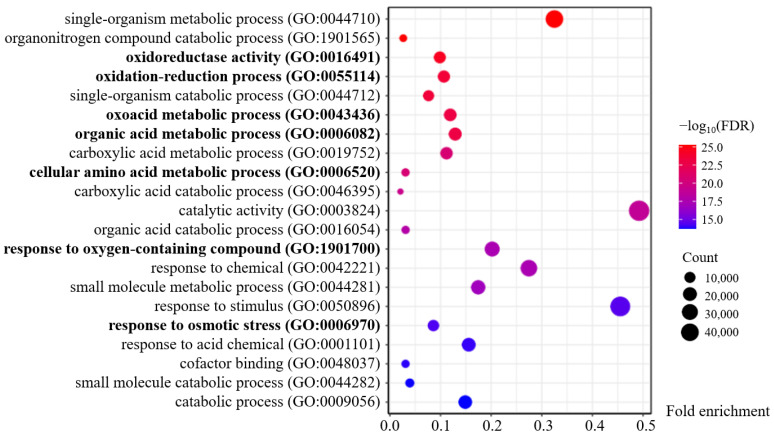
The main GO enrichment terms of the Turquoise module. Terms related to oxidation–reduction or organic metabolism are displayed in bold.

The Turquoise module with the most DEGs is significantly positively correlated with 48 h after salt treatment, which belongs to the growth recovery period. During this period, plant cells will reduce the accumulation of ROS induced by Na^+^ toxicity and synthesize a large number of organic compounds (proline, betaine, etc.) through hormone pathways such as abscisic acid and ethylene to increase cytoplasmic concentration and then alleviate damages caused by external high salt osmotic stress [3,4], which is consistent with the GO enrichment results of the Turquoise module (Figure 6, Table S3). The Turquoise module mainly involves biochemical reactions such as oxidative recovery (e.g., GO:0016491, GO:0055114, GO:1901700) and organic metabolism (e.g., GO:0043436, GO:0006082, GO:0006520, GO:0006970) (Figure 6).

The salt tolerance candidate genes *TaAAP* and *TaBAM* belong to the Turquoise module. One previous study has reported that a wheat AAP family member TaAAP1 can promote ethylene synthesis and upregulate the ethylene signaling pathway genes, ultimately improving seed salt-tolerance during germination [14]. The *TaAAP* identified in our study matches *TaAAP47* of wheat AAP family, and may also have similar salt tolerance functions as *TaAAP1*. In addition, *IbBAM1.1* has been shown to positively regulate salt tolerance in sweet potato plants by tuning ROS homeostasis and osmotic balance [13]. Increased BAM activity was observed in oats [21] and wheat [22] after salt stress, but its regulatory mechanism has not been reported yet. The *TaBAM* identified in this study may regulate this process. Through sequence alignment, it was found that *TaBAM* is the reported wheat grain development gene *TaBAM5.1-A* [23], speculating that *TaBAM* may affect grain composition and tolerance to osmotic stress by dynamically regulating starch and maltose content. However, *TaBAM* and a predicted interacting gene *TaERC* (*TraesCS2B02G453400*) were significantly downregulated at 48 h after salt stress, indicating the possibility of a unique regulatory mechanism that requires further research in the future.

## 4. Materials and Methods

### 4.1. Plant Materials and NaCl Treatment

Wheat salt-tolerance breeding line CH7034 [24], bred by Shanxi Agricultural University (formerly Shanxi Academy of Agricultural Sciences), was used for RNA-Seq. A set of wheat germplasm containing 114 varieties [7] (Table S4) was used for association analysis and marker verification. The above materials were provided by the Shanxi Key Laboratory of Crop Genetics and Molecular Improvement.

Seeds were sterilized using 1% hydrogen peroxide and laid on Petri dishes with moist filter paper, the uniform seeds with about 3 cm long embryonic roots were selected and transferred to sterile plastic boxes containing 1/2 Hoagland’s culture solution in a growth chamber under a 22/16 °C (day/night) temperature regime and a 16/8 h (light/dark) photoperiod with 60% relative humidity. When the seedlings grew to the three-leaf stage, they were exposed to 250 mmol/L NaCl for salt-stress treatment [25].

### 4.2. RNA-Seq

CH7034 seedling leaves were collected with three biological replications after 0, 1, 6, 24, and 48 h after NaCl stress and frozen in liquid nitrogen. These leaf samples were then embedded into dry ice and send to Biomarker Technologies Co., Ltd. (Beijing, China) to construct cDNA libraries and perform transcriptome sequencing using the HiSeq4000 platform (Illumina, San Diego, CA, USA). Clean reads were obtained by removing low quality reads that containing adapters and poly-N (>10%) or with a quality score <30 from the raw data, and mapped to the Chinese Spring reference genome (RefSeq v1.0, http://wheat-urgi.versailles.inra.fr/, accessed on 19 March 2024). The transcript level of each gene was measured with fragments per kilobase of exon model per million mapped fragments (FPKM) values, and genes with FPKM values below 1 in all samples were defined as non-expressed genes. Differential expression analysis between the control (0 h of NaCl treatment) and salt-stress groups (1, 6, 24, and 48 h of NaCl treatment) was performed using DESeq R package [26], with a threshold of |log2FoldChange| ≥ 1 and FDR ≤ 0.01.

### 4.3. WGCNA

WGCNA was constructed using R (version 4.3.1) software and the WGCNA package (version 1.72) for the leaf RNA-Seq data as previously described [7]. In brief, the transcript levels of all of the DEGs were converted into a similarity matrix and transformed to a topological overlap matrix using a parameter β value of 12. Genes with similar expression patterns were categorized into different modules using a bottom-up algorithm with a module minimum size cutoff of 30. The correlation between module eigengenes and the timepoints for salt stress was calculated using a Pearson test, and the individual modules with *p* < 0.05 were considered significantly correlated with the time-course. DEGs with a WGCNA edge weight >0.4 in the selected module were visualized using Cytoscape 3.7.2 software. In addition, DEGs of the Turquoise module were used for GO enrichment analysis on the BMKCloud platform (www.biocloud.net, accessed on 6 June 2024), and the categories or other detailed information on GO terms were listed in Table S3.

### 4.4. Association Analysis

Following the method described in Section 4.1, 114 wheat germplasms were subjected to salt stress for 7 days. Then, the leaf salt injury index (LSI) of each germplasm was investigated. Information on biallelic SNPs in the gene region was downloaded from the WheatUnion database (http://wheat.cau.edu.cn/WheatUnion/, accessed on 24 June 2024), with the search parameters being a minimum allele frequency of 0.1 and a maximum missing rate of 0.1. Based on 114 wheat germplasm resources, the correlation between the LSI data and DEGs was evaluated using association analysis in the R (version 4.3.1) software with the GAPIT package (version 3). The correlation was considered significant when −log_10_(*p*) > 4 (i.e., *p* < 0.0001).

### 4.5. KASP

Leaves of 114 wheat germplasm were collected during the three-leaf stage, and total DNA was extracted using an improved CTAB method. Kompetitive Allele Specific PCR (KASP) primers were designed for SNPs (Table 2). The KASP reaction system consists of 10 μL: 2 × KASP Master Mix (Aijixi Technology, Shanghai, China) 5.0 μL, DNA template (30 ng/μL) 4.85 μL, and primer mixture (100 pmol/μL) 0.15 μL. The KASP reactions were performed on a QuantStudio 3 Real-time PCR System (Applied Biosystems, Carlsbad, CA, USA) by running the following program: denature at 94 °C for 10 min, 10 cycles of touchdown PCR (94 °C for 20 s; touchdown at 60 °C initially and decreasing by −0.6 °C per cycle for 60 s), and 40 additional cycles (94 °C for 20 s; 55 °C for 60 s). PCR products were calculated in a fluorescence scanner under FAM and HEX channels. Microsoft Excel 2010 software was used for data processing and plotting, *t*-test was used to analyze the significance of differences between the two genotypes of KASP marker, with *p* < 0.05 considered significant and *p* < 0.01 considered extremely significant.

## 5. Conclusions

A total of 13,435 DEGs were obtained in the leaves of wheat salt-tolerant line CH7034 after salt stress. Based on these DEGs, nine modules containing 4029 DEGs related to time-courses were identified. Through association analysis and KASP marker validation, nine candidate genes associated with leaf salt injury index were further screened.

## Figures and Tables

**Table 1:** SNPs that significantly correlated with leaf salt injury index.

SNP-ID	Position (IWGSC v1.0)	*p* Value	Gene	Module	Annotation
1B28382[G/C]	chr1B:357643623	6.16 × 10^−5^	*TraesCS1B02G199700*	Turquoise	Aldo/keto reductase
1B28384[T/C]	chr1B:357643624	6.16 × 10^−5^			
1B62422[G/A]	chr1B:658447582	9.44 × 10^−5^	*TraesCS1B02G433700*	Red	B12D protein
2A19680[C/T]	chr2A:202051233	8.23 × 10^−5^	*TraesCS2A02G215100*	Turquoise	β-amylase
2A56718[T/C]	chr2A:735005679	1.18 × 10^−5^	*TraesCS2A02G508900*	Magenta	3-phosphoglycerate dehydrogenase
2A56735[C/G]	chr2A:735005886	1.18 × 10^−5^			
2A56900[C/T]	chr2A:735008442	3.74 × 10^−5^			
2A57026[T/C]	chr2A:735010697	7.21 × 10^−5^			
2A57040[C/T]	chr2A:735010824	3.20 × 10^−6^			
2A57350[C/T]	chr2A:735247227	2.77 × 10^−6^	*TraesCS2A02G509600*	Purple	Kinase
2B36776[G/A]	chr2B:394319058	2.21 × 10^−5^	*TraesCS2B02G286300*	Yellow	Senescence regulator S40
2B53697[G/A]	chr2B:583672455	6.16 × 10^−5^	*TraesCS2B02G410100*	Blue	Pentatricopeptide repeat-containing protein
2D16208[G/A]	chr2D:30979221	5.59 × 10^−5^	*TraesCS2D02G073700*	Brown	Germin-like protein
2D16220[C/G]	chr2D:30979584	1.13 × 10^−5^			
2D30145[T/C]	chr2D:112552049	9.02 × 10^−5^	*TraesCS2D02G168600*	Black	Transcription factor WRKY
3A25691[A/G]	chr3A:418765923	2.19 × 10^−5^	*TraesCS3A02G223700*	Brown	Chitinase domain-containing protein 1
3A25693[G/A]	chr3A:418765932	2.19 × 10^−5^			
3A25695[C/A]	chr3A:418765937	2.19 × 10^−5^			
3A25696[T/C]	chr3A:418765938	2.19 × 10^−5^			
3D34988[A/G]	chr3D:569527094	6.89 × 10^−5^	*TraesCS3D02G465600*	Yellow	Hydroxyethylthiazole kinase
5B14295[C/T]	chr5B:381844970	8.23 × 10^−5^	*TraesCS5B02G211000*	Yellow	Jasmonate zim-domain protein
6B00507[C/T]	chr6B:18731508	1.25 × 10^−5^	*TraesCS6B02G031700*	Yellow	Acid β-fructofuranosidase
6B00509[G/A]	chr6B:18731516	1.25 × 10^−5^			
7B00634[A/C]	chr7B:23439766	5.42 × 10^−5^	*TraesCS7B02G024500*	Red	Glutamyl-tRNA reductase
7B00635[A/C]	chr7B:23439791	3.77 × 10^−5^			
7B00636[G/C]	chr7B:23439798	3.77 × 10^−5^			
7B00758[C/T]	chr7B:23442344	9.84 × 10^−5^			
7D14857[T/C]	chr7D:141558991	2.17 × 10^−5^	*TraesCS7D02G189000*	Turquoise	Amino acid permease
7D14966[T/A]	chr7D:141564019	7.20 × 10^−5^			
7D14967[C/A]	chr7D:141564020	7.20 × 10^−5^			

**Table 2:** Sequences of KASP primers used in this study.

Marker Name	Primer Sequence (5′−3′)
KASP-1B62422-F1	GAAGGTGACCAAGTTCATGCTCCCCTCTGCTAGTTGGCC
KASP-1B62422-F2	GAAGGTCGGAGTCAACGGATTCCCCTCTGCTAGTTGGCT
KASP-1B62422-R	GAACATGGGAGGGATGGGTG
KASP-2A19680-F	GGCGTGTATGATGGATGTGC
KASP-2A19680-R1	GAAGGTGACCAAGTTCATGCTATGCCAGCGGATGTAGCC
KASP-2A19680-R2	GAAGGTCGGAGTCAACGGATTATGCCAGCGGATGTAGCT
KASP-2A56900-F	GCAAAACTCTTGCTATCCTTGGG
KASP-2A56900-R1	GAAGGTGACCAAGTTCATGCTGGGGCATATGCAATGAGATAAAGTC
KASP-2A56900-R2	GAAGGTCGGAGTCAACGGATTGGGGCATATGCAATGAGATAAAGTT
KASP-2B36776-F	GACTAGTGCAGCGGAGTAG
KASP-2B36776-R1	GAAGGTGACCAAGTTCATGCTATTCGCCCTCTTATTTCTGTTTC
KASP-2B36776-R2	GAAGGTCGGAGTCAACGGATTATTCGCCCTCTTATTTCTGTTTT
KASP-2B53697-F1	GAAGGTGACCAAGTTCATGCTGGTGGACTCGTGTCTTCGC
KASP-2B53697-F2	GAAGGTCGGAGTCAACGGATTGGTGGACTCGTGTCTTCGT
KASP-2B53697-R	TGTCCCATAGGAGGCAATGT
KASP-2D30145-F	GTAGGGCGAGAGGGGAGTC
KASP-2D30145-R1	GAAGGTGACCAAGTTCATGCTGCCTCCTAAGTCCAACACATGT
KASP-2D30145-R2	GAAGGTCGGAGTCAACGGATTGCCTCCTAAGTCCAACACATGC
KASP-5B14295-F1	GAAGGTGACCAAGTTCATGCTGCCAACTCCCTCACTCTTATACTC
KASP-5B14295-F2	GAAGGTCGGAGTCAACGGATTGCCAACTCCCTCACTCTTATACTT
KASP-5B14295-R	GACGCGGAGAGGGCATTTGC
KASP-7B00758-F	GGAGTTGGTCAACAAATTTCCG
KASP-7B00758-R1	GAAGGTGACCAAGTTCATGCTCAAGAGAAACAAGCTTTCCTGC
KASP-7B00758-R2	GAAGGTCGGAGTCAACGGATTCAAGAGAAACAAGCTTTCCTGT
KASP-7D14857-F	GGAATGGCCTTGATTGACTC
KASP-7D14857-R1	GAAGGTGACCAAGTTCATGCTGCCGCCTTCATTCTGGTTAAA
KASP-7D14857-R2	GAAGGTCGGAGTCAACGGATTGCCGCCTTCATTCTGGTTAAG

## Data Availability

Data are contained within the article and Supplementary Materials.

## Supplementary Materials

The following supporting information can be downloaded at: https://www.mdpi.com/article/10.3390/plants13182642/s1, Figure S1: Distribution of SNPs involved in the association analysis on wheat chromosomes; Table S1: Summary of RNA-sequencing results; Table S2: The modules and 4519 clustered genes in WGCNA; Table S3: GO enrichment of the Turquoise module; Table S4: 114 wheat germplasms used in association analysis.
