# Supplementary material for: Identification of Salt-Stress-Responding Genes by Weighted Gene Correlation Network Analysis and Association Analysis in Wheat Leaves
Source: Plants (Basel). 2024 Sep 21;13(18):2642. doi: 10.3390/plants13182642 (PMC11435117; doi:10.3390/plants13182642)
Supplement: Supplementary file 1 [file plants-13-02642-s001.zip › plants-3193071-figures.pdf]

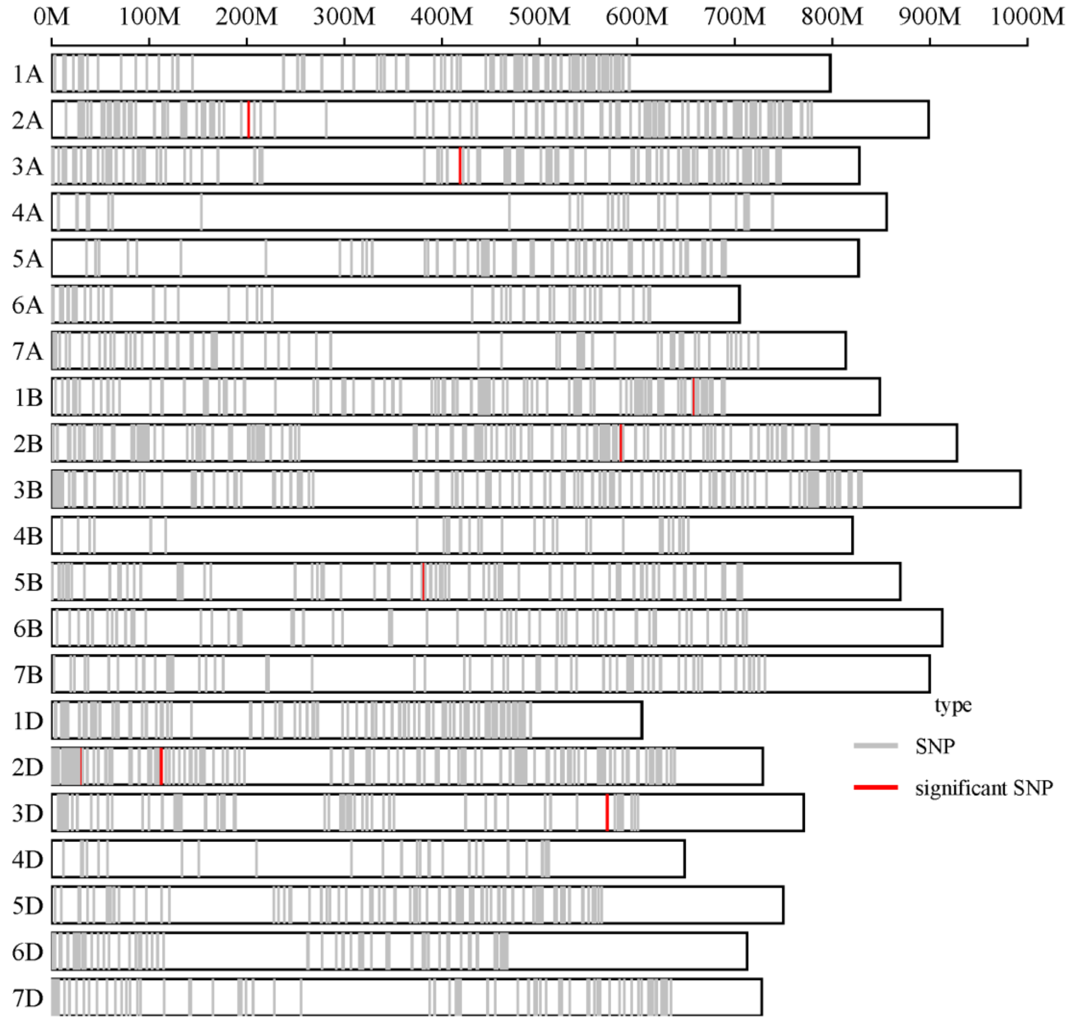

Figure S1. Distribution of SNPs involved in the association analysis on wheat chromosomes. The significant SNPs with −log10 (*p*)>4 are displayed in red.
